# Preparation and Characterization of an Anticancer Peptide from Oriental Tonic Food *Enteromorpha prolifera*

**DOI:** 10.3390/foods11213507

**Published:** 2022-11-04

**Authors:** Xiaosi Lin, Le Dong, Qingdan Yan, Yibo Dong, Li Wang, Fang Wang

**Affiliations:** 1 Fujian Province Key Laboratory for the Development of Bioactive Material from Marine Algae, Quanzhou Normal University, Quanzhou 362000, China; 2 College of Oceanology and Food Science, Quanzhou Normal University, Quanzhou 362000, China; 3 College of Food and Biological Engineering, Jimei University, Xiamen 361021, China

**Keywords:** peptide, anticancer, *Enteromorpha prolifera*, papain

## Abstract

*Enteromorpha prolifera* (*E. prolifera*), a tonic food in East Asian countries, is frequently studied for their pharmaceutical and healthcare applications. However, limited research has focused on antitumor peptides derived from this edible seaweed. In this study, we aimed to investigate the anticancer properties of peptides isolated from the hydrolysate of *E. prolifera* generated by a plethora of proteases including trypsin, papain, bromelain, and alkaline protease. The results showed that the hydrolysate produced by papain digestion exhibited remarkably stronger anticancer activity and was subjected to further purification by ultrafiltration and sequential chromatography. One heptapeptide, designated HTDT-6-2-3-2, showed significant antiproliferation activity towards several human cancer cell lines. The IC_50_ values for NCI-H460, HepG2, and A549 were 0.3686 ± 0.0935 mg/mL, 1.2564 ± 0.0548 mg/mL, and 0.9867 ± 0.0857 mg/mL, respectively. Moreover, results from flow cytometry confirmed that cell apoptosis was induced by HTDT-6-2-3-2 in a dose-dependent manner. The amino acid sequence for this heptapeptide, GPLGAGP, was characterized by Edman degradation and further verified by Liquid Chromatography-Tandem Mass Spectrometry. In silico analysis results suggested that XIAP could be a potential target for HTDT-6-2-3-2. Molecular docking simulation showed that HTDT-6-2-3-2 could occupy a shallow pocket in the BIR3 domain of XIAP, which is involved in the inhibitory effect of caspase-9 activation. In conclusion, this *E. prolifera* derived peptide exhibited strong anticancer properties, which could be explored for pharmaceutical applications.

## 1. Introduction

Cancer is among the leading causes of mortality worldwide. The incidence was estimated to be approximately 1,918,030, with 609,360 deaths in 2022 in the United States [1]. Traditionally, cancer could be treated with surgery, radiotherapy, or chemotherapy. With the advent of precision medicine, some types of cancer, such as non-small cell lung cancer (NSCLC), could be treated by targeted therapy or immunotherapy [2,3]. Although targeted therapy significantly reduces adverse effects as compared with chemotherapy and contributes to a decline in mortality, the driver gene mutations that are targetable remain to be characterized in most cancer types. As for immunotherapy, the clinical outcome depends on the infiltration and activation of T cells into the tumor microenvironment [4]. Consequently, some cancers, considered “cold” tumors, characterized by scarce tumor-infiltrating T cells, would not benefit from immune checkpoint therapy [5]. Therefore, it is imperative to expand our list of therapeutic options.

*Enteromorpha prolifera* (O.F.Müll.) J.Agardh, a type of edible green algae as well as an oriental folk medicine, belongs to the phylum *Chlorophyta*, class *Chlorophyceae*, order *Ulvales*, and genus *Enteromorpha*. It has attracted a lot of interest in anticancer research over the past decades. The original study found that a methanol-soluble extract of *E. prolifera* had antimutagenic and antitumor promotion properties [6]. Later, pheophytin was characterized as an active component with anticarcinogenic activity in vitro and in vivo [7,8]. Recently, a sulfated glucurono-xylo-rhamnan (EP-3-H) was purified from *E. prolifera*. This polysaccharide showed potent cytotoxicity to human lung cancer A549 cells and attenuated xenograft tumor growth in BALB/c-nude mice. Such effects could be attributed partially to the interaction between EP-3-H and fibroblast growth factors [9]. In another study, polysaccharides extracted from *Ulva* (*Enteromorpha*) *prolifera* O.F. Müller were reported to suppress H_2_O_2_-mediated MAPK activation and inhibit cell invasion by decreasing MMP-9 levels [10]. However, no research has been focused on the anticancer properties of peptides derived from *E. prolifera*. 

It has been widely accepted that peptides hold great potential in cancer treatment for their unique properties. For instance, most essential biological processes are controlled by protein-protein interactions (PPIs), and peptides are able to inhibit PPIs that are difficult to interrupt by small molecules [11]. Another advantage of peptides as potential therapeutics was that, unlike proteins and antibodies, peptides are smaller in molecular mass and have better tissue/cell penetration. Finally, peptide therapeutics are less likely to cause drug resistance because they are not dependent on flow pumps [12]. For these merits, several peptide drugs have been approved for cancer treatment including Leuprolide, Goserelin and Octreotide [13]. In this mechanism, anticancer peptides may exert their functions by destroying the structure of the cell membrane, inducing apoptosis, inhibiting angiogenesis or immune regulation [14].

Recently, some anticancer peptides have been isolated from dietary algae such as *Chlorella vulgaris*, *Chlorella pyrenoidosa*, and *Spirulina platensis* [15,16,17]. These discoveries prompt us to explore the anticancer properties of peptides derived from *E. prolifera*. In this study, a protein extract of *E. prolifera* was subjected to enzymatic hydrolysis using papain. Peptides with anticancer activity were fractionated by tangential-flow ultrafiltration and separated further by chromatography. One of the peptides, designated as HTDT-6-2-3-2, exhibited strong anti-proliferation activity on NCI-H460, HepG2, and A549 human cancer cell lines. The peptide sequence was determined by Edman degradation and further verified by Liquid Chromatography-Tandem Mass Spectrometry (LC-MS/MS). Additionally, the potential targets of HTDT-6-2-3-2 were predicted by in silico analysis. The possible mechanism of the apoptosis-inducing property was further explored by molecular docking. 

## 2. Materials and Methods

### 2.1. Reagents

*E. prolifera* was acquired from Zao Yi Jia Food Co., Ltd. (Ningde, China). Trypsin, papain, bromelain, and alkaline protease were from Hefei Bomei Biotechnology Co., Ltd. (Hefei, China); acetonitrile, High Performance Liquid Chromatography (HPLC) grade, was from Spectrum Chemical Mfg. Corp., (New Brunswick, NJ, USA); trifluoroacetic acid (TFA) was from Rhawn (Shanghai, China); DMSO was from Sigma-Aldrich (Shanghai, China); RPMI-1640 and DMEM medium were from Cytiva (Marlborough, MA, USA); fetal bovine serum (FBS) was from AusgeneX (Molendinar, QLD, Australia); Cell Counting Kit-8 (CCK-8) was obtained from Beyotime Biotechnology (Shanghai, China); and Annexin V-FITC Apoptosis Detection Kit was from KeyGEN BioTECH (Nanjing, China).

### 2.2. Preparation of Algae Powder

*E. prolifera* strips were dried at 60 °C for 4 h and then they were powdered with 80 mesh of particle size. An ultrasonication-assisted depigmentation process was performed. In brief, algae powder was suspended in 75% *v*/*v* ethanol in a glass Erlenmeyer flask (Sichuan Shubo (Group) Co., Ltd, Chongzhou, China), and incubated in the water bath of an ultrasonic cleaner (Beijing Usun United Science&Technology Co., Ltd, Beijing, China) (480 W, 40 kHz) for 30 min. The algae powder was precipitated by centrifugation (11,500× *g*, 10 min), and the supernatant was discarded. The depigmentation process was repeated once, as described above. The sediment was spread into the enamel dish and allowed to dry.

### 2.3. Protein Extraction

Protein extraction was performed as described previously with the following modification [18]. Briefly, 5.0 g of algae powder was mixed with 40 mL of pure water. The pH of the suspension was adjusted to 9 with 1.0 M NaOH, supplemented with pure water to a total volume of 50 mL, and incubated at 60 °C in a water bath for 2 h. After centrifugation at 11,500× *g* for 10 min, the supernatant was collected. The sediment was further extracted with 10 mL of pure water. After centrifugation, the resultant supernatants from these two sequential extractions were combined, and the pH was adjusted to 7 with 1.0 M HCl.

### 2.4. Protein Hydrolysis 

To generate peptides with different sizes and biological activities, we chose a panel of proteases that exhibit different recognition sites and are frequently used in the production of bioactive peptides [19,20]. An aliquot of 2 mL of protein extract was subjected to enzymic digestion using trypsin, papain, bromelain, or alkaline protease, respectively. All reactions were carried out at 55 °C for 2 h with an enzyme/substrate (E/S) ratio of 3000 U/g. For papain, bromelain, and trypsin digestion, we used pH 7 for reaction, while for alkaline protease, the pH for reaction was 8. The hydrolysates were then incubated at 100 °C for 3 min to inactivate the proteases, and the enzyme that produced the hydrolysate with the most potent cytotoxicity was chosen for further investigation.

For single-factor experiments, an aliquot of 2 mL of protein extract was used in each group. To test the effect of different E/S ratios on the cytotoxicity of the resulting hydrolysates, protein extract was digested with papain at 55 °C at pH 7 for 2 h with a series of E/S ratios ranging from 325 to 6000 U/g, respectively. For investigation of the impact of pH, the reaction was performed with an E/S ratio of 3000 U/g at 55 °C for 2 h with various pH as indicated. As to the influence of temperature for enzymic digestion, the reaction was performed with an E/S ratio of 3000 U/g at pH 7 for 2 h under different incubation temperatures as indicated.

To optimize the best parameters for large scale preparation of hydrolysates with potent anticancer activity, an L_9_(3^4^) orthogonal array study was conducted. The following parameters have been studied: E/S ratio, temperature, and pH, at three levels as indicated.

### 2.5. Large Scale Preparation of Anticancer Peptide

The hydrolysate was vacuum concentrated, and 2 volumes of ethanol were added to remove polysaccharide and protein. After centrifugation at 11,500× *g* for 10 min, the supernatant was collected and lyophilized. Then, the peptides were dissolved in pure water and fractionated using tangential flow filtration units with molecular weight cut-off (MWCO) for 2 kDa, 5 kDa, and 10 kDa, respectively. The resultant peptide fractions were tested for their cytotoxicity towards the NCI-H460 cell line. For peptide fractions with remarkable cytotoxicity, further chromatographic purification was performed sequentially by size-exclusive chromatography (SEC) and preparative reverse-phase HPLC. For HPLC purification, we used a Waters Atlantis T3 4.6 × 250 mm, 5 μm column (Waters, Shanghai, China), and a Waters Atlantis T3 20 × 250 mm column (Waters, Shanghai, China) for analytical and preparative applications, respectively. The gradient from solvent A (0.05% TFA in acetonitrile) to solvent B (0.05% TFA in de-ionized water) was varied from 5 to 70% of solvent A over 45 min at a flow rate of 10 mL/min and monitored at 210 nm.

### 2.6. Cell Culture and Cell Growth Inhibition Assay

The HepG2, A549, and NCI-H460 cell lines were purchased from the National Collection of Authenticated Cell Cultures (NCACC). HepG2 (Serial No. SCSP-510) and A549 (Serial No. SCSP-503) were maintained in DMEM media containing 10% FBS, while NCI-H460 (Serial No. SCSP-584) was cultured in RPMI-1640 media plus 10% FBS at 37 °C with 5% CO_2_.

For the cell growth inhibition assay, 4 × 10^3^ cells in 100 µL of medium were seeded per well in 96-well plates. Twelve hours later, wells in the control group were replaced with fresh medium, while those in the treatment group were supplemented with fresh medium containing peptides at the indicated concentration. After another incubation for 48 h, the cell growth inhibition rate was measured using the CCK-8 assay, following the instructions from the user’s manual. The inhibition rate was calculated as follows: 

Inhibition rate = [1 − (At − Ab)/(Ac − Ab)] × 100%, where At = absorbance value of the treatment group, Ac = absorbance value of the control group, and Ab = absorbance value of the blank respectively. The IC_50_ value was calculated by the modified Karber method.

### 2.7. Flow Cytometry

After treatment with HTDT-6-2-3-2 for 24 h, NCI-H460 cells were digested with 0.25% trypsin and washed twice with PBS. A total of 5×10^5^ cells were suspended in 500 μL of binding buffer and stained with Annexin V-FITC/Propidium Iodide (Jiangsu KeyGEN BioTECH Co., Ltd., Nanjing, China) for 15 min according to the manufacturer’s instructions. The labeled cells were examined by a Beckman Coulter CytoFLEX, and the percentage of apoptotic cells (lower right and upper right regions of the scatter plots) was calculated.

### 2.8. De Novo Sequencing of Peptide by Edman Degradation Assay and Mass Spectrometry Verification

The Edman degradation assay was conducted by Bio-Tech Pack Technology (Beijing, China). LC–MS/MS analysis was conducted by Applied Protein Technology (Shanghai, China). A Q Exactive mass spectrometer (Thermo Scientific, Shanghai, China) that was coupled to Easy nLC (Proxeon Biosystems, now Thermo Fisher Scientific, Shanghai, China) was used for mass spectrometry (MS) data analysis. The mass spectrometer was operated in positive ion mode. The theoretical molecular mass of the heptapeptide was calculated via the online tool Compute pI/MW (https://web.expasy.org/compute_pi/, accessed on 2 August 2022) [21,22]. 

### 2.9. Characterization of HTDT-6-2-3-2

The 3D structure of HTDT-6-2-3-2 was predicted by the PEP-FOLD3 server (https://bioserv.rpbs.univ-paris-diderot.fr/services/PEP-FOLD3/, accessed on 23 October 2022) [23]. For molecular target prediction, the amino acid sequence of HTDT-6-2-3-2 was converted to a SMILES string using the PepSMI online tool (https://www.novoprolabs.com/tools/convert-peptide-to-smiles-string, accessed on 20 October 2022), and the resulting SMILES string was used for target prediction by SwissTargetPrediction (http://www.swisstargetprediction.ch/, accessed on 17 October 2022) [24].

### 2.10. Molecular Docking

The crystal structure of XIAP was obtained from the Protein Data Bank (PDB ID: 5M6E). Water and the original ligand were removed, and molecular docking was performed via Molecular Operating Environment (MOE) 2019.0102 (Chemical Computing Group, Montreal, QC, Canada). The best docking model of HTDT-6-2-3-2 on the structure of XIAP was displayed.

## 3. Results

### 3.1. Hydrolysate of E. prolifera from Papain Digestion Exhibits Remarkable Inhibitory Activity on Cancer Cell Proliferation

**Figure 1:**
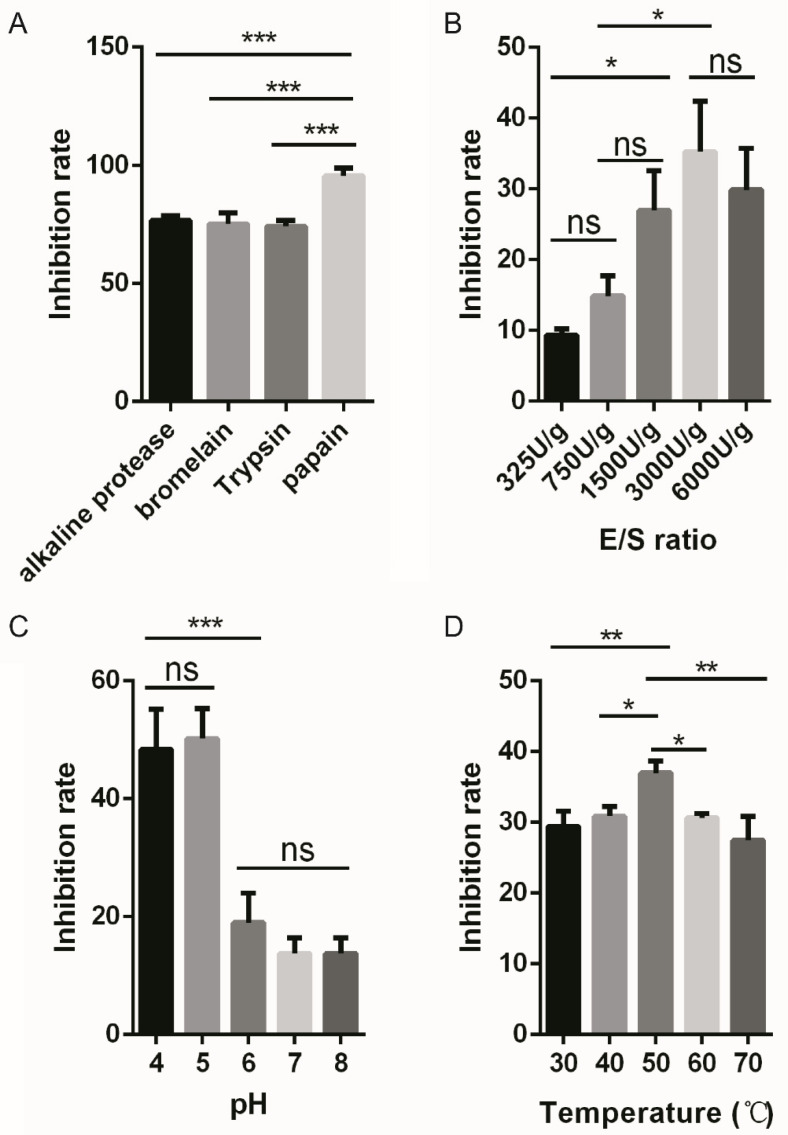
Protein hydrolysate of *E. prolifera* exhibit remarkable inhibition on cancer cell proliferation. (**A**) Hydrolysates generated using the indicated proteases showed differential anti-cancer activities. (**B**–**D**) Single factor experiments showed the impact of enzyme/substrate (E/S) ratio, pH, and temperature on the anti-proliferation property of hydrolysates generated by papain digestion, respectively. * *p* < 0.05, ** *p* < 0.01, *** *p* < 0.001 and ns, not significant (n = 3).

To prepare an anticancer peptide from *E. prolifera*, we began with the selection of a protease. A plethora of proteases, including papain, bromelain, alkaline protease, and trypsin, were investigated. After digestion, the hydrolysates were tested for cell growth inhibition via the CCK-8 assay. As shown in Figure 1A, hydrolysate prepared using papain exhibited the strongest inhibition on NCI-H460 cell growth to the degree of 95.56%, which is significantly higher than that of other enzymes. Therefore, papain was chosen for further study.

Next, we sought to explore the optimal condition of digestion mediated by papain via single factor experiments. Firstly, we tested the impact of the E/S ratio on the cell growth activity of hydrolysate. As shown in Figure 1B, treatment with papain using an E/S ratio ranging from 1500 to 6000 U/g significantly improves the cytotoxicity of the hydrolysate as compared to a lower E/S ratio such as 325 U/g, with an inhibition rate of 35.22% in the 3000 U/g group. Secondly, the influence of pH was analyzed. Under acidic pH conditions, such as pH 4 or 5, hydrolysates inhibit cell proliferation more effectively. At pH 5, the corresponding hydrolysate exhibited the strongest inhibition on cell growth to the extent of 50.18% (Figure 1C). A third factor that may play a role in the preparation of anticancer peptides is reaction temperature. In our experiment, the optimal temperature was 50 °C, and the corresponding hydrolysate displayed a growth inhibition rate of 36.94% (Figure 1D).

### 3.2. Optimization of Hydrolytic Parameters Using Papain

In order to perform large scale preparation of bioactive peptide with anticancer capacity, we optimized the parameters for papain digestion via a L_9_(3^4^) orthogonal array study based on the results of the aforementioned single factor experiments. The factors and levels are listed in Table 1.

In the orthogonal test, nine experiments were performed in duplicate to assess the impact of different combinations of E/S, pH, and temperature. As shown in Table 2, the R range data indicates that the factor order affecting cell inhibition rate was E/S > pH > temperature, with R range values of 17.99, 12.18, and 3.95, respectively. The optimal combination is A3B1C2, i.e., an E/S ratio of 4500 U/g, reaction pH 4.5, and digestion temperature at 50 °C. To confirm the optimal combination of parameters generated from the orthogonal test, enzymic hydrolysis under such conditions was conducted, and the corresponding hydrolysate showed a growth inhibition ratio of 61.74% on NCI-H460 cells, which surpassed that of hydrolysates obtained under conditions as described in Figure 1. Therefore, an E/S ratio of 4500 U/g, pH 4.5, and digestion temperature of 50 °C were chosen for the large-scale preparation of anticancer peptides from *E. prolifera* using papain digestion. 

### 3.3. Fractionation and Purification of Anticancer Peptide

**Figure 2:**
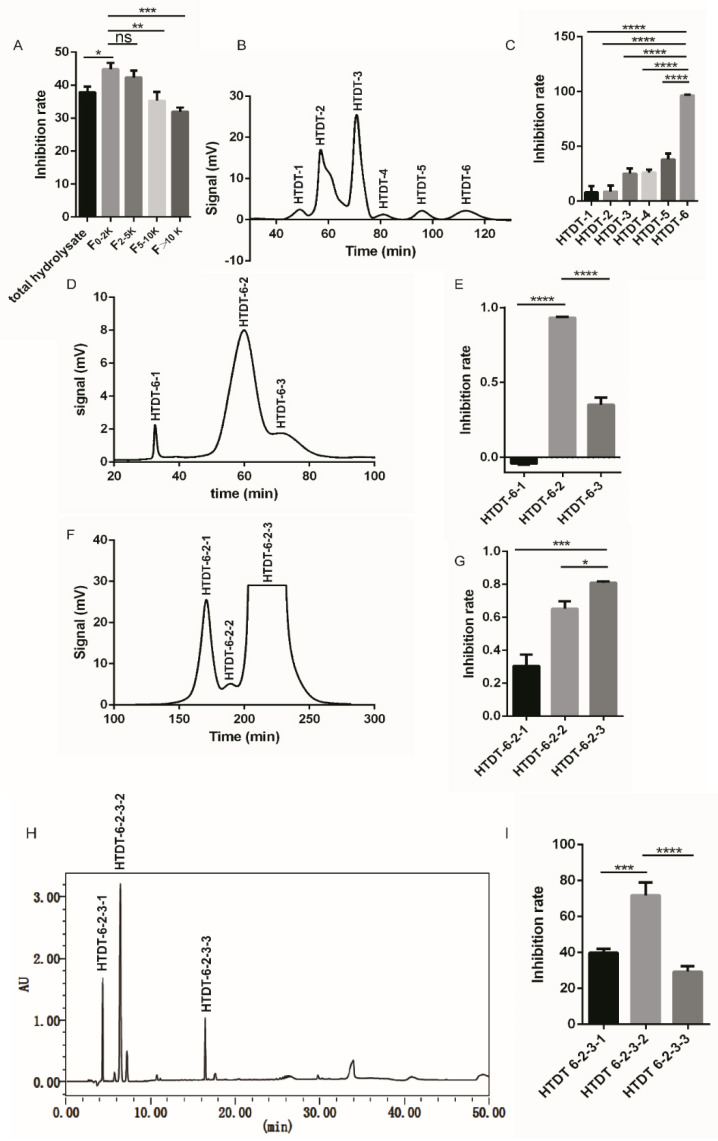
Purification of anticancer peptide by ultrafiltration and sequential chromatography. (**A**) After being fractionated by a series of tangential flow filtration units, peptide fractions with a molecular weight < 2 KDa (F_0-2K_) exhibit the most potent anti-proliferation activities. (**B**) The elution curve of F_0-2K_ by a 4 × 60 cm Sephadex LH-20 column using deionized water as the mobile phase. (**C**) Growth inhibition rate on NCI-H460 cells of the corresponding fractions shown in B. (**D**) The elution curve of HTDT-6 by 4 × 60 cm Sephadex LH-20 column using 20% (*v*/*v*) ethanol as mobile phase. (**E**) Growth inhibition rate of corresponding fractions showed in D. (**F**) The elution curve of HTDT-6-2 by a 1.5 × 170 cm Sephadex LH-20 column using 5% (*v*/*v*) ethanol as the mobile phase. (**G**) Growth inhibition rate of corresponding fractions showed in F. (**H**) HPLC profile of HTDT-6-2-3 separated by a Waters Atlantis T3 4.6 × 250 mm, 5 μm column. (**I**) Growth inhibition rate of the corresponding fractions shown in H. * *p* < 0.05, ** *p* < 0.01, *** *p* < 0.001, **** *p* < 0.0001 and ns, not significant (n = 3).

After large-scale preparation of hydrolysates, we intended to isolate peptides that exhibit strong cytotoxicity to cancer cells. To achieve this, we began with a fractionation assay using a series of tangential flow filtration units with MWCO for 2 kDa, 5 kDa, and 10 kDa, respectively. The hydrolysates were ultrafiltrated to obtain 4 fractions F_0–2K_, F_2–5K_, F_5–10K_, and F_>10K_, and then 3 mg/mL of each fraction was analyzed for its anticancer activity. As shown in Figure 2A, peptide fraction F_0–2K_ possessed an inhibition rate of 44.80% on the proliferation of NCI-H460, which ranked the highest in all groups analyzed, including total hydrolysate and peptide fractions with a larger molecular weight range. Therefore, F_0-2K_ is further purified by sequential chromatography. Firstly, it was separated by a 4 × 60 cm Sephadex LH-20 (Cytiva, Marlborough, MA, USA) column using deionized water as the mobile phase at a speed of 5.5 mL/min. The elution curve showed F_0-2K_ was separated into six fractions, namely HTDT-1–HTDT-6, respectively (Figure 2B). As shown in Figure 2C, HTDT-6 posed the most potent growth inhibition on NCI-H460 cells to the extent of 96.50 % at 1 mg/mL. Subsequently, HTDT-6 was further separated by a 4 × 60 cm Sephadex LH-20 column using 20% (*v*/*v*) ethanol as mobile phase at a speed of 10 mL/min, and the resultant peptide pools were split into 3 fractions: HTDT-6-1, HTDT-6-2, and HTDT-6-3 (Figure 2D). Among these, HTDT-6-2 was highly cytotoxic with an inhibition rate of 93.23% at 0.8 mg/mL (Figure 2E). Then, separation of HTDT-6-2 by a 1.5 × 170 cm Sephadex LH-20 column using 5% (*v*/*v*) ethanol as the mobile phase at a speed of 1 mL/min was conducted. Three fractions: HTDT-6-2-1, HTDT-6-2-2, and HTDT-6-2-3 were obtained, among which HTDT-6-2-3 displayed inhibitory activity on NCI-H460 with a rate of 80.87% at 0.8 mg/mL (Figure 2F,G). 

Finally, the constituents of HTDT-6-2-3 were analyzed by HPLC. As shown in Figure 2H, there were three major peaks after HPLC separation. Components corresponding to peaks 1–3, designated as HTDT-6-2-3-1, HTDT-6-2-3-2, and HTDT-6-2-3-3 respectively, were purified using a preparative HPLC system equipped with a Waters Atlantis T3 20×250 mm column. Among these, HTDT-6-2-3-2 showed the strongest cytotoxicity towards the NCI-H460 cell line, with an inhibition rate of 71.70% at 0.5 mg/mL (Figure 2I).

### 3.4. Cytotoxicity of Peptide HTDT-6-2-3-2 on Cancer Cell Lines 

**Figure 3:**
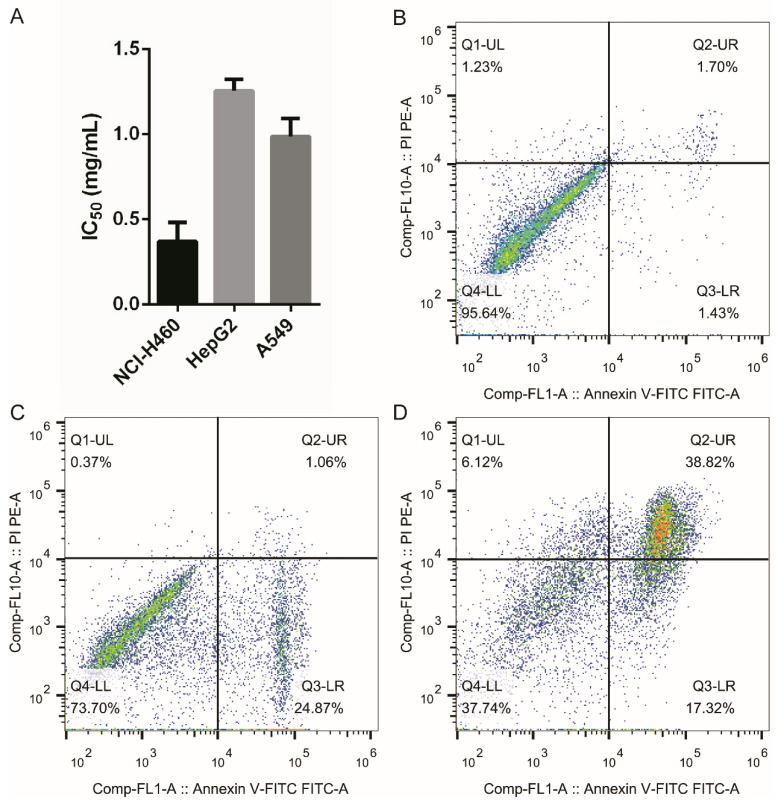
HTDT-6-2-3-2 induced cell death in a dose-dependent manner. (**A**) IC_50_ values of HTDT-6-2-3-2 on three cancer cell lines were determined. The result showed HTDT-6-2-3-2 exhibited strong anti-proliferation activity on NCI-H460, HepG2, and A549 human cancer cell lines, with an IC_50_ value of 0.3686 ± 0.0935 mg/mL, 1.2564 ± 0.0548 mg/mL, and 0.9867 ± 0.0857 mg/mL, respectively. (**B**–**D**) Analysis of apoptosis of NCI-H460 induced by HTDT-6-2-3-2 via flow cytometry. (**B**) control, (**C**) 1/2 IC_50_ of HTDT-6-2-3-2, and (**D**) IC_50_ of HTDT-6-2-3-2.

Because HTDT-6-2-3-2 inhibited the growth of NCI-H460 cells significantly, the IC_50_ values for various cell lines, including NCI-H460, HepG2, and A549, were determined. In our research, NCI-H460 was most sensitive to HTDT-6-2-3-2 with an IC_50_ of 0.3686 ± 0.0935 mg/mL, while HepG2 and A549 were more resistant to HTDT-6-2-3-2, with IC_50_ of 1.2564 ± 0.0548 mg/mL and 0.9867 ± 0.0857 mg/mL, respectively (Figure 3A).

To explore the underlying mechanism of cytotoxicity posed by HTDT-6-2-3-2, NCI-H460 cells treated with the indicated concentration of HTDT-6-2-3-2 for 24 h were labeled with Annexin-V FITC/PI and analyzed by flow cytometry. Treatment with HTDT-6-2-3-2 at 1/2 IC_50_ significantly induced apoptosis in about 25.93% of cells (Q2 + Q3, as shown in Figure 3C). Interestingly, when treated with HTDT-6-2-3-2 at IC_50_, approximately 56.14% of NCI-H460 cells suffered from apoptosis, comprising of 17.32% of early apoptotic and 38.82% of late apoptotic events, as illustrated in the Q2 and Q3 regions in Figure 3D, respectively. These results suggest that HTDT-6-2-3-2 induces potent cell death in a dose-dependent manner.

### 3.5. Characterization of HTDT-6-2-3-2

**Figure 4:**
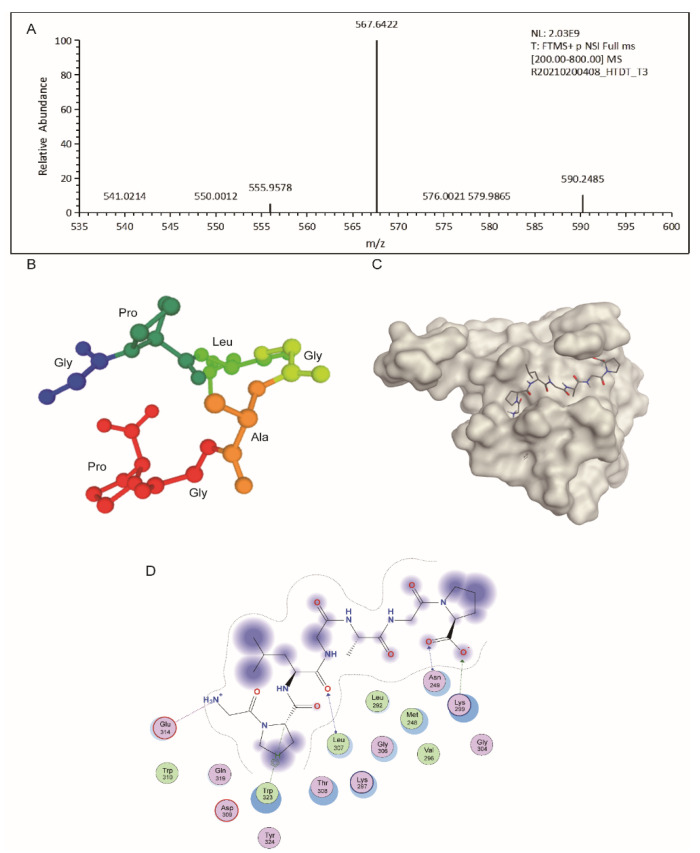
Characterization of HTDT-6-2-3-2. (**A**) MS spectrum result suggested that HTDT-6-2-3-2 bears a m/z of 567.6422, which matched perfectly with the calculated molecular weight of GPLGAGP heptapeptide. (**B**) The three-dimensional structure of HTDT-6-2-3-2 is predicted by PEP-FOLD3. (**C**) Molecular docking simulation of HTDT-6-2-3-2 binding to XIAP showed that HTDT-6-2-3-2 (shown as sticks) could be docked into a shallow pocket in the BIR3 domain of XIAP (gray). (**D**) Interaction between HTDT-6-2-3-2 and XIAP. Asn249, Lys299, Leu307, Glu314, and Trp323 from XIAP were involved in the association with HTDT-6-2-3-2.

The amino acid sequence of HTDT-6-2-3-2 was determined by the Edman degradation assay to be GPLGAGP, with a theoretical molecular weight of 567.64 Da. This is further verified by LC–MS/MS analysis, as we obtained a major peak with m/z = 567.6422, which fitted the calculated molecular weight of HTDT-6-2-3-2 perfectly (Figure 4A). There are two proline residues arranged in the second and last positions of this heptapeptide. As proline is a potent breaker for both α-helix and β-sheet structure, we assumed that HTDT-6-2-3-2 would exhibit neither an α-helix nor a β-sheet folding pattern [25]. This assumption was verified by the three-dimensional structure calculated by the PEP-FOLD3 online tool, showing that HTDT-6-2-3-2 displayed a random coil folding pattern (Figure 4B). As shown in Figure 3, HTDT-6-2-3-2 could induce apoptosis in the NCI-H460 cell line. Therefore, it is intriguing to explore the potential target for it. To achieve this, the amino acid sequence of HTDT-6-2-3-2 was converted to a SMILES string that was used to predict the potential target for HTDT-6-2-3-2 by the SwissTargetPrediction webtool. The top five targets ranked by probability are listed in Table 3. Interestingly, XIAP, encoding an intrinsic inhibitor of caspases, was predicted to be the most possible target for HTDT-6-2-3-2 [26]. Therefore, it is intriguing to hypothesize that HTDT-6-2-3-2 may disrupt the heterodimer formation between XIAP and caspases and, consequently, activate corresponding caspases and initiate cellular apoptosis. Molecular docking was performed to test the hypothesis. As shown in Figure 4C, HDTD-6-2-3-2 could be docked into a pocket in the BIR3 domain of XIAP, where it interacted with Asn249, Lys299, Leu307, Glu314, and Trp323 from XIAP (Figure 4D). 

## 4. Discussion

Serving as both food and medicine, the anticancer activity of *E. prolifera* has been investigated for decades. The anti-carcinogenesis properties have been attributed to pheophytin and polysaccharides [8,9,10]. In contrast, there have been no reports on the bioactive peptides derived from *E. prolifera*. In this study, we performed a large-scale enzymatic hydrolysis of protein extract from *E. prolifera* mediated by papain. The resulting peptide mixture was further fractionated via TFF and separated by sequential chromatography. One of the peptides, designated HTDT-6-2-3-2, exhibited potent anticancer activity towards the NSCLC cell line NCI-H460 with an IC_50_ value of 0.3686 ± 0.0935 mg/mL. To our knowledge, this is the first report on an anticancer peptide derived from *E. prolifera*. In this mechanism, treating with HTDT-6-2-3-2 at a concentration of half the IC_50_ remarkably induced apoptosis in NCI-H460 cells after 24 h of incubation. In contrast, at the concentration of IC_50_, cell death was further increased, although the proportion of cells suffering from early apoptosis decreased significantly in this circumstance. These results suggest that at higher concentration, HTDT-6-2-3-2 could induce apoptosis at an earlier time point post-treatment, and the increment in the amount of Annexin-V/PI double-positive cells may represent late apoptosis. In accordance with the assumption that HTDT-6-2-3-2 could induce apoptosis in cancer cells, XIAP was predicted to be at the top of a list of potential targets for HTDT-6-2-3-2. Molecular docking simulations show that HTDT-6-2-3-2 may occupy a shallow pocket in the BIR3 domain of XIAP. Of note, this domain is reported to be involved in the inhibition of caspase-9 activation as demonstrated by the crystal structure of caspase-9 in an inhibitory complex with XIAP-BIR3 [27]. Therefore, it is possible that HTDT-6-2-3-2 may prevent XIAP from attaching to caspase-9, consequently, triggering apoptotic cell death.

There are only a few reports available on anticancer peptides derived from algae. It has been shown that a hendecapeptide separated from hydrolysates of *Chlorella vulgaris* possesses a strong antiproliferation property against AGS cells with an IC_50_ value of 70.7 ± 1.2 μg/mL (72 h post treatment) [15]. Another peptide displaying tumor growth inhibitory activity was isolated from papain hydrolysates of *Chlorella pyrenoidosa*. Although its amino acid sequence was not determined, the IC_50_ value against HepG2 was about 426 μg/mL (72 h post treatment) [16]. A new peptide derived from *Porphyra haitanensis* hydrolysate after trypsin digestion shows strong inhibition against MCF-7 and HepG2 with IC_50_ values of 200.97 μg/mL and 276.85 μg/mL, respectively [28]. It is worth noting that IC_50_ values determined at 72 h post treatment are always lower than those determined at 48 h. Therefore, HTDT-6-2-3-2 reported here shows similar antiproliferation properties to those mentioned above. 

Interestingly, two more potential targets for HTDT-6-2-3-2 as predicted by SwissTargetPrediction were ACE and DPP4. The former was recognized as a pivotal target for treating hypertension; the latter was involved in the regulation of insulin levels and, consequently, a desirable target in treating type 2 diabetes mellitus (T2DM) [29,30]. As both hypertension and T2DM are increasing global threats to human health, a lot of research has been focused on finding inhibitors for ACE or DPP4 [18,19,31,32]. Our research suggested that HTDT-6-2-3-2 may also be novel inhibitors for ACE and DPP4. Future experiments will be conducted to verify this assumption. 

In conclusion, we report here a novel heptapeptide with potent anticancer activity and provide a detailed protocol for the large-scale preparation of this peptide from papain mediating enzymic hydrolysis. The amino acid sequence of this peptide is determined to be GPLGAGP by Edman degradation, which allows us to prepare this anticancer peptide by chemical synthesis or genetic engineering. This heptapeptide could induce apoptosis of cancer cells, probably due to its interaction with XIAP, which liberates caspase-9 from the inhibitory complex with XIAP and facilitates its activation. We also reported that ACE4 and DPP4 may be potential targets for HTDT-6-2-3-2, which may broaden its pharmaceutical application. Our research lays the foundation for high-value exploitation of *E. prolifera*.

## Figures and Tables

**Table 1:** L_9_(3^4^) orthogonal array design for papain hydrolysis.

Level	Factor		
	A, E/S (U/g)	B, pH	C, Temperature (°C)
1	2000	4.5	45
2	3000	5	50
3	4500	5.5	55

**Table 2:** Orthogonal array experimental results for papain hydrolysis.

Trial Number	Factor				Replication	
	A	B	C	Blank	X%	Y%
1	1	1	1	1	40.36	41.15
2	1	2	2	2	39.17	37.14
3	1	3	3	3	35.31	32.34
4	2	1	2	3	56.53	57.96
5	2	2	3	1	42.43	42.48
6	2	3	1	2	34.17	35.66
7	3	1	3	2	57.22	56.53
8	3	2	1	3	60.98	59.45
9	3	3	2	1	48.96	50.25
K1	37.58	51.63	45.30	44.27		
K2	44.87	46.94	48.34	43.32		
K3	55.57	39.45	44.39	50.43		
R range	17.99	12.18	3.95	7.11		
Factor order	A > B > C					
Priority level	A3B1C2					

**Table 3:** List of top five potential molecular targets of HTDT-6-2-3-2 as predicted by SwissTargetPrediction.

Target	Common Name	Uniprot ID	chEMBL ID	Target Class	Probability
Inhibitor of apoptosis protein 3	XIAP	P98170	CHEMBL4198	Other cytosolic protein	0.76
Angiotensin- converting enzyme	ACE	P12821	CHEMBL1808	Protease	0.65
Dipeptidyl peptidase IV	DPP4	P27487	CHEMBL284	Protease	0.50
HLA class I histocompatibility antigen A-3	HLA-A	P04439	CHEMBL2632	Surface antigen	0.29
Beta-secretase 1	BACE1	P56817	CHEMBL4822	Protease	0.14

## Data Availability

Data will be made available upon reasonable request.
